# Healthcare resource utilization and costs among patients with heart failure with preserved, mildly reduced, and reduced ejection fraction in Spain

**DOI:** 10.1186/s12913-022-08614-x

**Published:** 2022-10-08

**Authors:** Carlos Escobar, Beatriz Palacios, Luis Varela, Martín Gutiérrez, Mai Duong, Hungta Chen, Nahila Justo, Javier Cid-Ruzafa, Ignacio Hernández, Phillip R. Hunt, Juan F. Delgado

**Affiliations:** 1 Cardiology Department, University Hospital La Paz, Madrid, Spain; 2 AstraZeneca Farmaceutica, Madrid, Spain; 3 Evidera, London, UK; 4 AstraZeneca, Gaithersburg, MD USA; 5 Evidera, Stockholm, Sweden; Karolinska Institute, Department of Neurobiology, Care Sciences, and Society, Stockholm, Sweden; 6 Evidera, Barcelona, Spain; 7 Atrys Health, Barcelona, Spain; 8 Cardiology Department, University Hospital 12 de Octubre, CIBERCV, Madrid, Spain

**Keywords:** Cost, Heart failure, Healthcare resource utilization, Hospitalization, Sacubitril/valsartan, SGLT2 inhibitors

## Abstract

**Aims:**

To describe healthcare resource utilization (HCRU) of patients with heart failure with preserved (HFpEF), mildly reduced (HFmrEF), and reduced ejection fraction (HFrEF) in Spain.

**Methods:**

Adults with ≥ 1 HF diagnosis and ≥ 1 year of continuous enrolment before the corresponding index date (1/January/2016) were identified through the BIG-PAC database. Rate per 100 person-years of all-cause and HF-related HCRU during the year after the index date were estimated using bootstrapping with replacement.

**Results:**

Twenty-one thousand two hundred ninety-seven patients were included, of whom 48.5% had HFrEF, 38.6% HFpEF and 4.2% HFmrEF, with the rest being of unknown EF. Mean age was 78.8 ± 11.8 years, 53.0% were men and 83.0% were in NYHA functional class II/III. At index, 67.3% of patients were taking renin angiotensin system inhibitors, 61.2% beta blockers, 23.4% aldosterone antagonists and 5.2% SGLT2 inhibitors. Rates of HF-related outpatient visits and hospitalization were 968.8 and 51.6 per 100 person-years, respectively. Overall, 31.23% of patients were hospitalized, mainly because of HF (87.88% of total hospitalizations); HF hospitalization length 21.06 ± 17.49 days (median 16; 25th, 75th percentile 9–27). HF hospitalizations were the main cost component: inpatient 73.64%, pharmacy 9.67%, outpatient 9.43%, and indirect cost 7.25%. Rates of all-cause and HF-related HCRU and healthcare cost were substantial across all HF subgroups, being higher among HFrEF compared to HFmrEF and HFpEF patients.

**Conclusions:**

HCRU and cost associated with HF are high in Spain, HF hospitalizations being the main determinant. Medication cost represented only a small proportion of total costs, suggesting that an optimization of HF therapy may reduce HF burden.

**Supplementary Information:**

The online version contains supplementary material available at 10.1186/s12913-022-08614-x.

## Introduction

Heart failure (HF) affects more than 60 million people all around the world (approximately 15 million in Europe and 6 million in United States) [1, 2]. The current prevalence of HF reaches 1–2% of the adult people in developed countries, and 8.52 per 1,000 inhabitants worldwide [1–3]. However, the prevalence of HF will increase in the following years, mainly due to the ageing of the population [1–6].

In spite of traditional HF treatments, mortality rates remain unacceptably high [1, 2, 7]. In addition, HF is the main cause of hospitalization in elderly people in Western countries and it is responsible for 1–2% of all hospitalizations [1, 2, 8]. In fact, one out of 6 patients with HF with reduced ejection fraction (HFrEF) will develop worsening HF within 18 months of initial diagnosis [9]. Consequently, it is expected that the number of HF hospitalizations will markedly increase in the future [1, 10]. Recent clinical trials have demonstrated that some drugs can positively modify the clinical course of HF, in both, HFrEF (i.e. sacubitril-valsartan and some sodium-glucose co-transporter-2 inhibitors [SGLT2i]) and HF with preserved EF (HFpEF) (i.e. some SGLT2i), leading to a reduction of HF burden [11–16].

HF is associated with huge direct and indirect costs, largely due to HF hospitalization, representing 1–2% of total healthcare costs in Europe and United States [17–19]. In addition, HF accounts for 9.91 million years lost due to disability (YLDs) and 346.17 billion US $ expenditure worldwide [3]. As a result, it is important to ascertain the main determinants of HF costs, in order to optimize the management of HF that may allow a reduction in HF costs [7, 18].

Although some studies have analyzed the clinical profile and management of HF stratified by EF (HFrEF, HF with mildly reduced EF [HFmrEF] and HFpEF) [20–25], there are very few studies that have focused on identifying cost drivers according to HF phenotype, particularly in Spain [26–32].

This study aimed to describe healthcare resource utilization (HCRU) and direct medical costs including HF-related and all-cause outpatient visits, hospitalizations, specialist visits, and poly-pharmacy, stratified by EF subgroups, through the analysis of a nationally representative Spanish database.

## Methods

Retrospective cohort study that included a prevalent cohort of adults with at least one inpatient or outpatient HF diagnosis, and at least one year of continuous enrolment before the corresponding index date (1 January 2016). Therefore, evaluation time for the analysis included data from 1 January 2016 through 31 December 2016 (i.e. one year follow-up). Patients were excluded if they had less than one year of continuous enrolment before the index date, < 18 years at index date, or had chronic kidney disease stage V that required dialysis at any time before the index date.

Data were collected from the BIG-PAC database in Spain that includes secondary healthcare data of non-selected 1.8 million patients from primary care and hospital centers, across seven Autonomous Communities in Spain. Before export to BIG-PAC®, data were rigorously anonymized and dissociated. Costs were calculated using sources from the Spanish National Healthcare System. This study was conducted in accordance with the Declaration of Helsinki and approved by the Investigation Ethics Committee of HM Hospitales (Madrid, Spain). No informed consent was required in this study, as secondary data were used and all information was completely anonymized and dissociated from patients’ identity. Several studies have demonstrated representativeness of this database of the Spanish population and its ability to accurately determine the clinical profile, treatments, healthcare resource utilization and costs in Spain [4, 5, 18].

Clinical characteristics, including demographics, HF diagnosis, cardiovascular risk factors, vascular disease, chronic kidney disease by stage [33] and other comorbidities, as well as treatments were determined at baseline. Comorbidities were based on data any time up to the index date, unless otherwise specified. The International Classification of Diseases (ICD)-9 and ICD-10 codes (https://eciemaps.mscbs.gob.es) were considered for the diagnosis of HF and comorbidities (supplementary Table 1). Treatments during one year before index date were recorded from the registries for dispensing medicines, according to the Anatomical Therapeutic Chemical Classification System [34]. Data were stratified by EF subgroups, HFpEF: EF ≥ 50%; HFrEF: EF ≤ 40%; HFmrEF: EF > 40- < 50%; HF with unspecified EF (HFuEF): patients without an echocardiographic result at baseline.

During the year after the index date, HF-related hospitalizations, outpatient visits, costs as well as all-cause HCRU were estimated using cost data from the Spanish National Health Service, and included: inpatient (number of hospitalizations > 24 h, length of hospital stays, cost), outpatient (number of visits to general practitioners, the number of visits to the specialist, cost), emergency visits (number of visits to the emergency department, cost), pharmacy (total prescription cost for HF and non-HF medications), and indirect cost relating to work morbidity-induced productivity loss. Inpatient and outpatient visits with a HF ICD-10 code (supplementary Table 1), as the primary code were assumed to be HF-related HCRU.

## Statistical analysis

Baseline characteristics and treatments were summarized using descriptive statistics and stratified by EF subgroups. Qualitative variables were described by their absolute and relative frequency distributions. Measures of central tendency (mean, median), dispersion (standard deviation [SD], 25th, 75th percentile), and categories, where appropriate, were used to describe the quantitative variables. The rates of HCRU, overall and HF-related were estimated within the year after the index date, stratified by EF subgroups. The results were reported per 100 person-years. The confidence interval (CI) for HCRU was estimated using nonparametric bootstrapping method, with the number of resampling set at 1,000. Length of inpatient stays was estimated as mean (SD) and median (25th, 75th percentile). The number of prescriptions per patient was estimated as mean (SD) and median (25th, 75th percentile), and the proportion of patients with 0, 1, 2, 3, 4 or ≥ 5 prescriptions (polypharmacy) was also determined. HCRU costs, overall and HF-related were estimated as mean (SD) per patient. Results in the HFmrEF and HFpEF subgroups were compared with the HFrEF subgroup. To explore an association between continuous variables amongst EF subtypes, the two-sample t-test was used for variables normally distributed and the Mann–Whitney U test for those non-normally distributed. The chi-square test was used for categorical variables. A level of statistical significance of 0.05 was applied in all statistical tests. The CI for HCRU was estimated using nonparametric bootstrapping method (SciPy package). The data were analyzed using the statistical package SPSS v25.0 (SPSS Inc., Chicago, Illinois, USA).

## Results

A total of 21,297 patients with HF were included, of whom 48.5% had HFrEF, 38.6% HFpEF and 4.2% HFmrEF, with the rest being of unknown EF (Table 1). Overall, mean age was 78.8 ± 11.8 years, 53.0% were men and 83.0% were in New York Heart Association (NYHA) functional class II or III. Comorbidities were common at index (67.5% had hypertension, 38.2% coronary artery disease, 31.8% type 2 diabetes and 30.3% chronic kidney disease). Regarding HF drugs, 67.3% of patients were taking renin angiotensin system inhibitors, 61.2% beta blockers, 23.4% aldosterone antagonists and 5.2% SGLT2i. Compared with patients with HFrEF, patients with HFmrEF were older, more commonly women, presented more frequently with hypertension, dyslipidemia and atrial fibrillation, but less frequently with diabetes, coronary artery disease, peripheral artery disease and chronic obstructive pulmonary disease. Compared to those patients with HFrEF, patients with HFpEF were older, more commonly women, more patients were on NYHA functional class II, and presented more frequently with dyslipidemia and atrial fibrillation, but less frequently with coronary artery disease, chronic kidney disease, stroke, peripheral artery disease, chronic obstructive pulmonary disease and dementia. Regarding HF treatments, relative fewer patients with HFmrEF were taking diuretics, renin angiotensin system inhibitors, SGLT2i, digoxin and ivabradine than those patients with HFrEF. All HF drugs were more commonly taken by patients with HFrEF than by those with HFpEF. Among patients with HFpEF, the clinical profile and treatments did not differ according to EF (50 to < 60% vs ≥ 60%).

**Table 1:** Baseline clinical characteristics and treatments

	**HF cohort (*n* = 21,297; 100%)**	**HFrEF (*n* = 10,323; 48.5%)**	**HFmrEF (*n* = 903; 4.2%)**	**HFpEF (*n* = 8,225; 38.6%)**	**HFpEF (50 to < 60%) (*n* = 2,995; 14.1%)**	**HFpEF (≥ 60%) (*n* = 5,230; 24.6%)**	**HFuEF (*n* = 1,846; 8.7%)**	***p*-value (HFmrEF vs HFrEF)**	***p*-value (HFpEF vs HFrEF)**
**Biodemographic data**									
**Age (years) at index date**									
Mean (SD)	78.8 (11.8)	73.6 (9.7)	81.7 (9.9)	84.0 (11.4)	84.1 (11.3)	84.0 (11.4)	83.3 (11.9)	< 0.001	< 0.001
Median (25th, 75th percentile)	79.1 (71.9—87.6)	73.8 (67.5—79.8)	80.6 (75.6—89.4)	85.6 (78.2—91.9)	85.7 (78.4—89.4)	85.5 (78.2—91.9)	85.5 (78.0—91.9)		
Range (min—max)	(18—102.8)	(18—96.36)	(21.6—98.8)	(18.5—102.8)	(18.6—98.8)	(18.5—100.5)	(18.9—100.5)		
**Gender, male, n (%)**	11,278 (53.0)	6,782 (65.7)	440 (48.7)	3,068 (37.3)	1,102 (36.8)	1,966 (37.6)	988 (53.5)	< 0.001	< 0.001
**NHYA at index date, n (%)**									
Class I	2,137 (10.0)	1,030 (10.0)	91 (10.1)	817 (9.9)	322 (10.8)	495 (9.5)	199 (10.8)		
Class II	8,949 (42.0)	3,689 (35.7)	332 (36.8)	4,176 (50.8)	1,504 (50.2)	2,672 (51.1)	752 (40.7)	0.494	< 0.001
Class III	8,728 (41.0)	4,750 (46.0)	411 (45.5)	2,783 (33.8)	1,016 (33.9)	1,767 (33.8)	784 (42.5)		
Class IV	1,013 (4.8)	612 (5.9)	56 (6.2)	280 (3.4)	92 (3.1)	188 (3.6)	65 (3.5)		
Unknown	470 (2.2)	242 (2.3)	13 (1.4)	169 (2.1)	61 (2.0)	108 (2.1)	46 (2.5)		
**Cardiovascular risk factors, n (%)**									
Hypertension	14,379 (67.5)	6,885 (66.7)	662 (73.3)	5,550 (67.5)	2,021 (67.5)	3,529 (67.5)	1,282 (69.5)	< 0.001	0.261
Dyslipidemia	10,457 (49.1)	4,681 (45.4)	457 (50.6)	4,384 (53.3)	1,601 (53.5)	2,783 (53.2)	935 (50.7)	0.002	< 0.001
Diabetes type 1	844 (4.0)	499 (4.8)	32 (3.5)	258 (3.1)	100 (3.3)	158 (3.0)	55 (3.0)	0.080	< 0.001
Diabetes type 2	6,772 (31.8)	3,331 (32.3)	236 (26.1)	2,630 (32.0)	1,127 (37.6)	1,503 (28.7)	575 (31.2)	< 0.001	0.672
**Vascular disease, n (%)**									
Coronary artery disease	8,124 (38.2)	4,520 (43.8)	288 (31.9)	2,653 (32.3)	971 (32.4)	1,682 (32.2)	663 (35.9)	< 0.001	< 0.001
Chronic kidney disease	6,452 (30.3)	3,411 (33.0)	272 (30.1)	2,286 (27.8)	849 (28.4)	1,437 (27.5)	483 (26.2)	0.073	< 0.001
Stage Unknown	2,706 (12.7)	1,451 (14.1)	106 (11.7)	953 (11.6)	358 (12.0)	595 (11.4)	196 (10.6)		
Stage I	179 (0.8)	86 (0.8)	6 (0.7)	73 (0.9)	26 (0.9)	47 (0.9)	14 (0.8)		
Stage II	644 (3.0)	327 (3.2)	19 (2.1)	250 (3.0)	85 (2.8)	165 (3.2)	48 (2.6)		
Stage III	2,225 (10.5)	1,179 (11.4)	106 (11.7)	789 (9.6)	296 (9.9)	493 (9.4)	151 (8.2)		
Stage IV	524 (2.5)	281 (2.7)	26 (2.9)	153 (1.9)	63 (2.1)	90 (1.7)	64 (3.5)		
Stage V	174 (1.0)	87 (1.1)	9 (1.1)	68 (0.9)	21 (0.9)	47 (0.9)	10 (0.6)		
Myocardial Infarction	3,174 (14.9)	1,645 (15.9)	103 (11.4)	1,110 (13.5)	384 (12.8)	726 (13.9)	316 (17.1)	< 0.001	< 0.001
Stroke	2,254 (10.6)	1,327 (12.9)	107 (11.9)	617 (7.5)	297 (9.9)	320 (6.1)	203 (11.0)	0.385	< 0.001
Peripheral arterial disease	1,074 (5.0)	616 (6.0)	24 (2.7)	337 (4.1)	146 (4.9)	191 (3.7)	97 (5.3)	< 0.001	< 0.001
**Other comorbidities, n (%)**									
COPD	3,319 (15.6)	1,716 (16.6)	121 (13.4)	1,202 (14.6)	441 (14.7)	761 (14.6)	280 (15.2)	0.012	< 0.001
Atrial fibrillation	6,785 (31.9)	2,538 (24.6)	283 (31.3)	3,364 (40.9)	1,205 (40.2)	2,159 (41.3)	600 (32.5)	< 0.001	< 0.001
Anemia within 1 year before index date	6,540 (30.7)	3,266 (31.6)	255 (28.2)	2,503 (30.4)	910 (30.4)	1,593 (30.5)	516 (28.0)	0.035	0.078
Cancer before index date	2776 (13.0)	1,313 (12.72)	109 (12.1)	1,077 (13.1)	368 (12.3)	709 (13.6)	277 (15.0)	0.574	0.449
Dementia	1,058 (5.0)	568 (5.5)	45 (5.0)	360 (4.4)	168 (5.6)	192 (3.7)	85 (4.6)	0.510	< 0.001
**HF drugs, n (%)**									
Diuretics	15,780 (74.1)	7,759 (75.2)	649 (71.9)	5,964 (72.5)	2,174 (72.6)	3,790 (72.5)	1,408 (76.3)	0.029	< 0.001
ACEi/ARB	14,335 (67.3)	7,840 (76.0)	574 (63.6)	4,806 (58.4)	1,734 (57.9)	3,072 (58.7)	1,115 (60.4)	< 0.001	< 0.001
Beta-blockers	13,043 (61.2)	6,631 (64.2)	602 (66.7)	4,693 (57.1)	1,711 (57.1)	2,982 (57.0)	1,117 (60.5)	0.143	< 0.001
Aldosterone antagonists	4,973 (23.4)	2,609 (25.3)	207 (22.9)	1,765 (21.5)	654 (21.8)	1,111 (21.2)	392 (21.2)	0.118	< 0.001
Digoxin	4,311 (20.2)	2,307 (22.4)	162 (17.9)	1,437 (17.5)	526 (17.6)	911 (17.4)	405 (21.9)	0.002	< 0.001
Ivabradine	1,502 (7.1)	873 (8.5)	38 (4.2)	449 (5.5)	181 (6.0)	268 (5.1)	142 (7.7)	< 0.001	< 0.001
SGLT2i (among diabetics)	1,115 (5.2)	704 (6.8)	34 (3.8)	267 (3.3)	89 (3.0)	178 (3.4)	110 (6.0)	< 0.001	< 0.001
Hydralazine and nitrate	19 (0.1)	7 (0.1)	1 (0.1)	11 (0.1)	5 (0.2)	6 (0.1)	0	0.643	0.152
ARNI	0	0	0	0	0	0	0	–	–

All treatments were assessed within 12 months before index. Patients on combination drugs were counted in each respective treatment class. Therefore, each treatment class included patients on monotherapy and combination therapy. Lookback period for all comorbidities was any time before index date (event date < index date), unless otherwise specified; lookback period for all prescription was 12 months prior to index date

*Abbreviations*: *ACE* Angiotensin-converting enzyme, *ARB* Angiotensin receptor II blocker, *ARNI* Dual angiotensin receptor and neprilysin inhibition, *COPD* Chronic obstructive pulmonary disease, *HF* Heart failure, *HFmrEF* Heart failure with mildly reduced ejection fraction, *HFpEF* Heart Failure with preserved ejection fraction, *HFrEF* Heart Failure with reduced ejection fraction, *HFuEF* Heart Failure with unspecified ejection fraction, *NYHA* New York Heart Association, *SD* Standard deviation, *SGLT2i* Sodium-glucose co-transporter-2 inhibitors

All-cause and HF-related HCRU are presented in Table 2. Overall, rates of HF-related outpatient visits and hospitalization in the study year were 968.8 (95% confidence interval [CI] 962.1–975.7) and 51.6 (95% CI 50.3–52.9) 95% confidence interval [CI] 961.5–975.1) and 51.6 (95% CI 51.5–54.3) per 100 person-years, respectively. Visits to the general practitioner were 26.3 times more frequent than to the specialist. Rates of all-cause and HF-related HCRU were higher among patients with HFrEF compared to those with HFmrEF and HFpEF. HFmrEF rates were intermediate between HFrEF and HFpEF.

**Table 2:** Outpatient visits and hospitalization during the index year

	All HF patients (*n* = 21,297)			HFrEF (*n* = 10,323)			HFmrEF (*n* = 903)				HFpEF (*n* = 8225)			
	Patients (n)	Visits (n)	Rate^a^	Patients (n)	Visits (n)	Rate^a^	Patients (n)	Visits (n)	Rate^a^	P vs HFrEF	Patients (n)	Visits (n)	Rate^a^	P vs HFrEF
All-cause HCRU														
Outpatient Visits	21,290	305,498	1451.4 (1440.8–1461.9)	10,323	203,271	1995.5 (1981.4–2009.8)	902	9056	1010.9 (988.6–1036.2)	< 0.001	8219	75,080	922.9 (914.7–932.4)	< 0.001
GPs visits	21,259	286,227	1359.8 (1349.4–1370.0)	10,322	190,763	1872.7 (1858.1–1887.0)	899	8476	946.2 (924.4–970.9)	< 0.001	8193	69,957	859.9 (851.8–868.8)	< 0.001
Specialist visits	10,595	19,271	91.6 (90.0–93.0)	7038	12,508	122.8 (120.6–124.9)	303	580	64.7 (58.3–71.6)	< 0.001	2677	5123	63 (60.9—65.1)	< 0.001
Hospitalization	6652	13,087	62.2 (60.7—63.7)	3606	7699	75.6 (73.1–77.8)	270	480	53.6 (46.9–59.9)	< 0.001	2242	4025	49.5 (47.4—51.5)	< 0.001
HF-related HCRU														
Outpatient Visits	21,252	203,922	968.8 (962.1–975.7)	10,323	138,456	1359.2 (1350.2–1368.4)	899	5854	653.5 (638.8–670.2)	< 0.001	8190	47,899	588.8 (583.4—594.4)	< 0.001
GPs visits	21,127	196,447	933.3 (926.5–940.2)	10,320	131,372	1289.7 (1280.2–1299.2)	895	5818	649.5 (634.6–666.4)	< 0.001	8083	47,597	585.1 (579.5–590.8)	< 0.001
Specialist visits	6003	7475	35.5 (34.7—36.3)	5612	7084	69.5 (68.2–70.9)	36	36	4 (2.7–5.3)	< 0.001	302	302	3.7 (3.3–4.1)	< 0.001
Hospitalization	5846	10,852	51.6 (50.3—52.9)	3246	6058	59.5 (57.3–61.4)	230	389	43.4 (37.3–48.9)	< 0.001	1917	3609	44.4 (42.4–46.3)	< 0.001

	HFpEF (50 to <60%) (*n*=2995)			HFpEF (≥60%) (*n*=5230)			HFuEF (*n*=1846)		
	Patients (n)	Visits (n)	Rate^a^	Patients (n)	Visits (n)	Rate^a^	Patients (n)	Visits (n)	Rate^a^
All-cause HCRU									
Outpatient Visits	2992	27464	927.8 (913.5-941.8)	5227	47616	920.1 (909.9-930.2)	1846	18091	987.5 (971.1-1003.7)
GPs visits	2984	25643	866.3 (851.8-879.6)	5209	44314	856.3 (846.2 - 866,4)	1845	17031	929.6 (913.5-944.9)
Specialist visits	937	1821	61.5 (57.7-65.0)	1740	3302	63.8 (60.9-66.6)	577	1060	57.9 (53.6-62.0)
Hospitalization	814	1453	49.1 (45.8-52.6)	1428	2572	49.7 (47.3-52.2)	534	883	48.2 (44.2-52.2)
HF-related HCRU									
Outpatient Visits	2984	17572	593.6 (584.4-603.2)	5206	30327	586 (579.2-592.9)	1840	11713	639.4 (628.8-649.0)
GPs visits	2952	17483	590.6 (581.2-600.1)	5131	30114	581.9 (575.1-588.9)	1829	11660	636.5 (625.8-646.3)
Specialist visits	89	89	3 (2.3-3.6)	213	213	4.1 (3.6-4.6)	53	53	2.9 (2.1-3.6)
Hospitalization	695	1306	44.1 (40.9-47.5)	1222	2303	44.5 (42.2-47.0)	453	796	43.5 (39.3-47.5)

^a^Rate per 100 person-years (95% confidence interval)

*HF* Heart failure, *HFmrEF* Heart failure with mildly reduced ejection fraction, *HFpEF* Heart Failure with preserved ejection fraction, *HFrEF* Heart failure with reduced ejection fraction, *HFuEF* Heart Failure with unspecified ejection, *HCRU* Healthcare resource utilization

Overall, 31.23% of patients were hospitalized, mainly because of HF (87.88% of total hospitalizations). Mean duration of HF hospitalization was 21.06 ± 17.49 days (median 16; 25th, 75th percentile 9–27) and despite the elderly nature of these patients, 7.72% had recorded sick leave due to HF (mean 23.38 ± 7.85 days). Mean number of HF-related prescriptions in the follow-up year was 16.09 ± 7.77 and the majority of patients were polymedicated. A higher proportion of hospitalizations in patients with HFrEF were related to HF in compared with patients with HFmrEF and HFpEF (34.93% vs 29.90% and 27.26%, respectively; both *P* < 0.001). A higher proportion of hospitalizations in patients with HFrEF were related to HF compared with patients with HFmrEF and HFpEF (90.02% vs 85.19% and 85.50%, respectively). In addition, duration of HF hospitalization was higher among patients with HFrEF compared to those with HFmrEF and HFpEF (median 20; 25th, 75th percentile 13–36 days vs 14: 25th, 75th percentile 9–21.5 and 12; 25th, 75th percentile 6–21, respectively; both *P* < 0.001). The number and length of medical-absenteeism spells were higher in patients with HFrEF than in those with HFmrEF and HFpEF. Among patients with HFpEF, HCRU did not differ according to EF (50 to < 60% vs ≥ 60%) (Table 3).

**Table 3:** Healthcare resource utilization during the index year

	**All HF patients (*n* = 21,297)**	**HFrEF (*n* = 10,323)**	**HFmrEF (*n* = 903)**	**P vs HFrEF**	**HFpEF (*n* = 8225)**	**P vs HFrEF**	**HFpEF (50 to < 60%) (*n* = 2995)**	**HFpEF (≥ 60%) (*n* = 5230)**	**HFuEF (*n* = 1846)**
**Length of hospital stays (all-cause)**									
Number of patients hospitalized, n (%)	6652 (31.23)	3606 (34.93)	270 (29.90)	< 0.001	2242 (27.26)	< 0.001	814 (27.18)	1428 (27.30)	534 (28.93)
Mean (SD)	19.59 (17.73)	24.93 (20.3)	16.18 (13.52)		13.51 (11.31)		13.43 (11.27)	13.56 (11.33)	10.73 (8.41)
Median (25th, 75th percentile)	14 (7–25)	19 (10–35)	12 (7–19)		10 (5–18)		10 (5–18)	10 (5–19)	8 (5–15)
**Length of hospital stays (HF-related)**									
Number of patients hospitalized, n (%)	5846 (27.45)	3246 (31.44)	230 (25.47)	< 0.001	1917 (23.31)	< 0.001	695 (23.21)	1222 (23.37)	453 (24.54)
Mean (SD)	21.06 (17.49)	26.49 (19.61)	17.57 (13.38)		14.54 (11.34)		14.38 (11.23)	14.63 (11.4)	11.46 (8.33)
Median (25th, 75th percentile)	16 (9–27)	20 (13–36)	14 (9–21.5)		12 (6 -21)		12 (6–21)	12 (6–21)	9 (5–16)
**Number of prescriptions (all-cause)**									
Number of patients, n (%)	21,297 (100)	10,323 (100)	903 (100)	< 0.001	8225 (100)	< 0.001	2995 (100)	5230 (100)	1846 (100)
Mean (SD)	38.86 (13.29)	41.39 (13.12)	34.3 (12.17)		36.23 (12.97)		36.24 (13)	36.22 (12.95)	38.67 (13.32)
Median (25th, 75th percentile)	38 (29–47)	41 (32–50)	33 (26–42)		35 (27–45)		35 (27–44)	35 (27–45)	38 (29–48)
**Number of prescriptions (HF-related)**									
Number of patients, n (%)	21,297 (100)	10,323 (100)	903 (100)	< 0.001	8225 (100)	< 0.001	2995 (100)	5230 (100)	1846 (100)
Mean (SD)	16.09 (7.77)	16.74 (7.85)	13.96 (6.84)		15.47 (7.64)		15.43 (7.57)	15.5 (7.68)	16.16 (7.89)
Median (25th, 75th percentile)	15 (10–21)	16 (11–22)	13 (9–18)		15 (10–20)		15 (10–20)	15 (10–20)	15 (10–21)
**Work absences (number of days) (all-cause)**									
Number of patients, n (%)	3178 (14.92)	2011 (19.48)	93 (10.30)	< 0.001	871 (10.59)	< 0.001	321 (10.72)	550 (10.52)	203 (10.99)
Mean (SD)	17.81 (9.89)	21.74 (10.2)	11.58 (3.41)		10.79 (3.68)		10.86 (3.56)	10.75 (3.75)	11.92 (4.25)
Median (25th, 75th percentile)	15 (10–26)	23 (13–30)	12 (9–14)		11 (8–14)		11 (8–13)	11 (8–14)	13 (9–15)
**Work absences (number of days) (HF-related)**									
Number of patients, n (%)	1644 (7.72)	1283 (12.42)	43 (4.76)	< 0.001	255 (3.10)	< 0.001	93 (3.11)	162 (3.10)	63 (3.41)
Mean (SD)	23.38 (7.85)	26.82 (4.86)	12.19 (2.54)		10.51 (1.99)		10.69 (2.01)	10.41 (1.98)	13.16 (2.29)
Median (25th, 75th percentile)	24 (19–30)	27 (23–31)	13 (10–14)		11 (9–12)		11 (9–12)	10 (9–12)	13 (11–15)
**Polypharmacy (not only HF treatments), n (%)**									
1	20 (0.09)	5 (0.05)	0	< 0.001	14 (0.17)	< 0.001	4 (0.13)	10 (0.19)	1 (0.05)
2	91 (0.43)	50 (0.48)	2 (0.22)		35 (0.43)		12 (0.4)	23 (0.44)	4 (0.22)
3	369 (1.73)	128 (1.24)	19 (2.1)		193 (2.35)		71 (2.37)	122 (2.33)	29 (1.57)
4	1512 (7.1)	486 (4.71)	99 (10.96)		786 (9.56)		310 (10.35)	476 (9.1)	141 (7.64)
≥ 5	19.305 (90.65)	9654 (93.52)	783 (86.71)		7197 (87.49)		2598 (86.75)	4599 (87.93)	1671 (90.52)

*HF* Heart failure, *HFmrEF* Heart failure with mildly reduced ejection fraction, *HFpEF* Heart Failure with preserved ejection fraction, *HFrEF* Heart failure with reduced ejection fraction, *HFuEF* Heart Failure with unspecified ejection, *HRCU* Healthcare resource utilization, *SD* Standard deviation

Overall and HF-related healthcare resource costs per patient during the index year are presented in Tables 4 and 5. Overall and HF-related cost were 3193.2 ± 4457.7€ and 2518.8 ± 4323.8€ (78.88% of the total cost) per patient, respectively. Hospitalizations were the main component of healthcare resource costs: overall: inpatient 61.48%, pharmacy 18.42%, outpatient 11.67%, indirect cost 8.43%; HF-related: inpatient 73.64%, pharmacy 9.67%, outpatient 9.43%, indirect cost associated with medical absenteeism 7.25%. Overall and HF-related healthcare resource costs per patient were higher among HFrEF than HFmrEF and HFpEF patients. Among patients with HFpEF, overall and HF-related healthcare resource costs per patient did not differ according to EF (50 to < 60% vs ≥ 60%).

**Table 4:** Overall healthcare resource costs and per patient during the index year

	**All HF patients (*N* = 21,297)**				**All HFrEF (*N* = 10,323)**				**All HFmrEF (*N* = 903)**					**All HFpEF (*N* = 8225)**				
	n	Total cost (€)	€1	€2	n	Total cost (€)	€1	€2	n	Total cost (€)	€1	€2	P vs HFrEF	n	Total cost (€)	€1	€2	P vs HFrEF
**Outpatient**	21,297	7.938.396,6	372.7 (196)	372.9 (196)	10,323	5.268.083,97	510.3 (174)	510.3 (174)	903	235.708,44	261 (108.1)	261.3 (107.8)	< 0.001	8225	1.968.105,33	239.3 (108.1)	239.5 (107.9)	< 0.001
GPs visits^a^	21,297	6.637.604,1	311.7 (175.1)	312.2 (174.7)	10,323	4.423.793,97	428.5 (170.4)	428.6 (170.4)	903	196.558,44	217.7 (85.2)	218.6 (84.1)	< 0.001	8225	1.622.302,83	197.2 (85.7)	198 (85)	< 0.001
Specialist visits^a^	21,297	1.300.792,5	61.1 (73.7)	122.8 (57.8)	10,323	844.290,00	81.8 (74.2)	120 (59.1)	903	39.150,00	43.4 (69)	129.2 (55.4)	< 0.001	8225	345.802,50	42 (68.1)	129.2 (54.7)	< 0.001
**Inpatient**																		
Hospitalizations (> 24 hs)^b^	21,297	41.808.135,6	1963.1 (4311.6)	6285 (5688.3)	10,323	28.845.380,10	2794.3 (5419.3)	7999.3 (6514.7)	903	1.401.691,20	1552.3 (3356.8)	5191.4 (4338.4)	< 0.001	8225	9.722.949,10	1182.1 (2705.2)	4336.7 (3628.9)	< 0.001
**Pharmacy**																		
Prescriptions^c^	21,297	12.528.920,7	588.3 (206.3)	588.3 (206.3)	10,323	6.456.456,17	625.4 (204.8)	625.4 (204.8)	903	470.263,00	520.8 (186.3)	520.8 (186.3)	< 0.001	8225	4.516.345,35	549.1 (201.2)	549.1 (201.2)	< 0.001
**Indirect cost**																		
Cost of absence from work^d^	21,297	5.730.105,4	269.1 (749.7)	1803.1 (1000.5)	10,323	4.425.103,62	428.7 (983.5)	2200.4 (1032.8)	903	109.003,17	120.7 (373.1)	1172.1 (345.3)	< 0.001	8225	951.171,58	115.6 (357.2)	1092 (372.6)	< 0.001
**Total Overall cost**		68.005.558,3	3193.2 (4457.7)			44.995.023,9	4358.7 (5522.8)			2.216.665,8	2454.8 (3391.2)		< 0.001		17.158.571,4	2086.1 (2737.8)		< 0.001

	**All HFpEF (50 to < 60%) (*N* = 2995)**				**All HFpEF (≥ 60%) (*N* = 5230)**				**All HFuEF (*N* = 1846)**			
	n	Total cost (€)	€1	€2	n	Total cost (€)	€1	€2	n	Total cost (€)	€1	€2
**Outpatient**	2995	717.578,67	239.6 (108)	239.8 (107.8)	5230	1.250.526,66	239.1 (108.1)	239.2 (108)	1846	466.498,89	252.7 (103.5)	252.7 (103.5)
GPs visits^a^	2995	594.661,17	198.6 (85.6)	199.3 (84.9)	5230	1.027.641,66	196.5 (85.8)	197.3 (85)	1846	394.948,89	213.9 (79.3)	214.1 (79.2)
Specialist visits^a^	2995	122.917,50	41 (68.1)	131.2 (54.8)	5230	222.885,00	42.6 (68.1)	128.1 (54.6)	1846	71.550,00	38.8 (65.3)	124 (55.4)
**Inpatient**												
Hospitalizations (> 24 hs)^b^	2995	3.507.116,10	1171 (2688.2)	4308.5 (3616.4)	5230	6.215.833,00	1188.5 (2715.1)	4352.8 (3637.3)	1846	1.838.115,20	995.7 (2131.2)	3442.2 (2699.1)
**Pharmacy**												
Prescriptions^c^	2995	1.642.571,36	548.4 (201.4)	548.4 (201.4)	5230	2.873.773,99	549.5 (201.1)	549.5 (201.1)	1846	1.085.856,22	588.2 (206.4)	588.2 (206.4)
**Indirect cost**												
Cost of absence from work^d^	2995	352.716,85	117.8 (359.8)	1098.8 (360.4)	5230	598.454,73	114.4 (355.8)	1088.1 (379.8)	1846	244.826,99	132.6 (403.4)	1206 (430.1)
**Total Overall cost**		6.219.983,0	2076.8 (2731.1)			10.938.588,4	2091.5 (2741.9)			3.635.297,3	1969.3 (2203.8)	

^a^Cost was calculated based on standard cost for GP/ specialist visits

^b^Cost of inpatient stays was calculated based on the DRG-based reimbursement of the stays

^c^Cost was based on full price of product

^d^Cost of absence from work was calculated by multiplying the number of days of absence from work due to sickness by the mean daily salary of a working person

All cost were presented in euros

*€1* mean cost per patient (SD) accounting for all patients, *€2* mean cost per patient (SD) accounting for patients with ≥ 1 resource use, *GP* General practitioner, *HF* Heart failure, *HFmrEF* Heart failure with mildly reduced ejection fraction, *HFpEF* Heart Failure with preserved ejection fraction, *HFrEF* Heart failure with reduced ejection fraction, *HFuEF* Heart Failure with unspecified ejection, *HRCU* Healthcare resource utilization, *n* number of patients, *SD* Standard deviation

**Table 5:** HF-related healthcare resource costs per patient during the index year

	**All HF patients (*N* = 21,297)**				**All HFrEF (*N* = 10,323)**				**All HFmrEF (*N* = 903)**					**All HFpEF (*N* = 8225)**				
	n	Total cost (€)	€1	€2	n	Total cost (€)	€1	€2	n	Total cost (€)	€1	€2	P vs HFrEF	n	Total cost (€)	€1	€2	P vs HFrEF
**Outpatient**	21,297	5.060.168,4	237.6 (131.9)	238.1 (131.6)	10,323	3.524.686,7	341.4 (107.9)	341.4 (107.9)	903	137.349,4	152.1 (133)	152.8 (53.8)	< 0.001	8225	1.124.159,4	136.7 (56.4)	137.3 (55.9)	< 0.001
GPs visits^a^	21,297	4.555.605,93	213.9 (118)	215.6 (116.9)	10,323	3.046.516,68	295.1 (111.5)	295.2 (111.4)	903	134.919,42	149.4 (119.2)	150.7 (54.5)	< 0.001	8225	1.103.774,43	134.2 (58.2)	136.6 (55.9)	< 0.001
Specialist visits^a^	21,297	504.562,50	23.7 (41.3)	84.1 (31.1)	10,323	478.170,00	46.3 (48.5)	85.2 (31.8)	903	2.430,00	2.7 (41.8)	67.5 (0)	< 0.001	8225	20.385,00	2.5 (12.7)	67.5 (0)	< 0.001
**Inpatient**																		
Hospitalizations (> 24 hs)^b^	21,297	39.504.394,50	1854.9 (4212.4)	6757.5 (5614)	10,323	27.597.079,10	2673.4 (5295)	8501.9 (6293.9)	903	1.296.756,90	1436.1 (4248.3)	5638.1 (4294.6)	< 0.001	8225	8.945.087,50	1087.5 (2641.4)	4666.2 (3638.7)	< 0.001
**Pharmacy**																		
Prescriptions^c^	21,297	5.187.669,82	243.6 (119.2)	243.6 (119.2)	10,323	2.612.507,32	253.1 (120.6)	253.1 (120.6)	903	191.566,65	212.1 (119.7)	212.1 (104.6)	< 0.001	8225	1.929.884,67	234.6 (117.4)	234.6 (117.4)	< 0.001
**Indirect cost**																		
Cost of absence from work^d^	21,297	3.890.309,98	182.7 (669.1)	2366.4 (794.7)	10,323	3.482.130,05	337.3 (912.1)	2714.1 (492.4)	903	53.034,04	58.7 (680.9)	1233.3 (256.9)	< 0.001	8225	271.242,80	33 (187.8)	1063.7 (201.8)	< 0.001
**Total HF-related cost**		53.642.542,7	2518.8 (4323.8)			37.216.403,2	3605.2 (5379.6)			1.678.707,0	1859 (4361.4)		< 0.001		12.270.374,4	1491.8 (2650.6)		< 0.001

	All HFpEF (50 to < 60%) (*N* = 2995)				All HFpEF (≥ 60%) (*N* = 5230)				All HFuEF (*N* = 1846)			
	n	Total cost (€)	€1	€2	n	Total cost (€)	€1	€2	n	Total cost (€)	€1	€2
Outpatient	2995	411.438,3	137.4 (55.8)	137.9 (55.3)	5230	712.721,2	136.3 (56.8)	136.9 (56.2)	1846	273.972,9	148.4 (51.6)	148.9 (51)
GPs visits^a^	2995	405.430,77	135.4 (57.5)	137.3 (55.5)	5230	698.343,66	133.5 (58.5)	136.1 (56.1)	1846	270.395,40	146.5 (52.8)	147.8 (51.1)
Specialist visits^a^	2995	6.007,50	2 (11.5)	67.5 (0)	5230	14.377,50	2.7 (13.3)	67.5 (0)	1846	3.577,50	1.9 (11.3)	67.5 (0)
Inpatient												
Hospitalizations (> 24 hs)^b^	2995	3.207.395,50	1070.9 (2608.9)	4615 (3603.2)	5230	5.737.692,00	1097.1 (2660.1)	4695.3 (3659.8)	1846	1.665.471,00	902.2 (2062.8)	3676.5 (2673.1)
Pharmacy												
Prescriptions^c^	2995	699.218,57	233.5 (115.8)	233.5 (115.8)	5230	1.230.666,10	235.3 (118.3)	235.3 (118.3)	1846	453.711,18	245.8 (121.1)	245.8 (121.1)
Indirect cost												
Cost of absence from work^d^	2995	100.602,74	33.6 (191)	1081.7 (203.5)	5230	170.640,06	32.6 (185.9)	1053.3 (200.7)	1846	83.903,09	45.5 (245.6)	1331.8 (231.5)
Total HF-related cost		4.418.655,1	1475.3 (2622.6)			7.851.719,3	1501.3 (2666.8)			2.477.058,2	1341.9 (2083.1)	

^a^Cost was calculated based on standard cost for GP/ specialist visits

^b^Cost of inpatient stays was calculated based on the DRG-based reimbursement of the stays

^c^Cost was based on full price of product

^d^Cost of absence from work was calculated by multiplying the number of days of absence from work due to sickness by the mean daily salary of a working person

All cost were presented in euros

*€1* Mean cost per patient (SD), *€2* Mean cost per patient (SD) accounting for patients with ≥ 1 resource use, *GP* General practitioner, *HF* Heart failure, *HFmrEF* Heart failure with mildly reduced ejection fraction, *HFpEF* Heart Failure with preserved ejection fraction, *HFrEF* Heart failure with reduced ejection fraction, *HFuEF* Heart Failure with unspecified ejection, *HRCU* Healthcare resource utilization, *n* number of patients, *SD* Standard deviation

## Discussion

Our study showed in a wide sample of subjects with HF that HCRU and costs are substantial in Spain, HF hospitalizations being the main driver of healthcare cost. By contrast, medication cost represents only a small proportion of total costs, suggesting that the best way to decrease HF-related costs is to reduce the risk of HF hospitalization through the optimization of HF therapy. HFrEF is associated with higher HCRU and direct and indirect costs and a higher proportion of the total costs are attributable to HF compared with HFmrEF and HFpEF. Compared with previous studies, our data provided a very comprehensive view about the cost drivers of HF according to HF phenotype in Spain.

In our study, around half of patients had HFrEF, 40% HFpEF and 5% HFmrEF. Although some disparities in the numbers can be found across studies, as HFpEF is markedly associated with older age, our figures were in line with previous studies [21, 23, 24]. In fact, previous studies have shown that data obtained from the BIG-PAC database are completely up-to-date [4, 5, 18]. Notably, our study showed that there were relevant differences in the clinical profile of patients with HFrEF compared to those with HFmrEF or HFpEF, particularly related with age and the prevalence of comorbidities. Compared with HFrEF, patients with HFmrEF or HFpEF were older, more commonly women, with more atrial fibrillation, but less ischemic heart disease. These differences in the clinical profile between HF subgroups have also been observed by others [20, 21, 23–25]. As these differences may have an impact on the clinical course of patients with HF, it is important to ascertain whether HCRU and healthcare costs may vary according to the type of HF, as well. In this context, the information provided by our study may be of great significance.

With regard to HCRU, HF-related outpatient visits were very common (969 per 100 person-years). Despite that, many patients were not taking the appropriate disease-modifying treatment, as guidelines recommend [1]. More than 30% of patients did not have prescriptions for renin angiotensin system inhibitors or beta-blockers. These data suggest that although HF patients require a close follow-up, treatment is not adequately optimized. Of note, visits to the general practitioner were 26.3 times more frequent than to the specialist. In fact, it has been observed a marked increase of HF burden in primary care [35]. As a result, a better coordination between healthcare levels is necessary to improve the management of this population [36]. In this context, a higher use of telemonitoring technology and cardiology electronic consultations would be desirable, as this may improve coordination between primary care and specialists, by facilitating the dialogue and interaction between heath care levels [37, 38]. In addition, this may also facilitate appropriate care transitions between hospital and home, leading to a reduction of hospital readmission rates [39, 40]. Furthermore, this interaction should not be limited to physicians, but also to other healthcare professionals (i.e. pharmacists, nurses, social workers), achieving a greater comprehensive involvement of the interprofessional team, reducing the risk of adverse events [41, 42]. This is even more important during the vulnerable period after the acute event, either hospitalization, visit to the emergency department or the outpatient clinic/day hospital [43]. This period represents a real window of opportunity to improve the management of HF patients.

Our study showed that overall and HF-related cost were high (3193€ and 2519€, respectively), hospitalizations, particularly HF hospitalization being the main driver (approximately 75% of HF-related costs). Although with some differences in the numbers, previous studies have also shown that HF hospitalizations are the largest contributor to HF burden [18, 44, 45]. This is very important, as in recent years there has been an increase in HF hospitalizations [46, 47]. As a result, a greater use of disease-modifying therapies is warranted to reduce HCRU and HF-related costs [18]. Unfortunately, our study showed that these drugs are still underused in clinical practice and that there is still much room for improvement. However, considering the date our data were taking (2016), it is likely that current numbers will be higher [18].

Although HF-related costs were high in the whole HF cohort, our study showed that costs were higher among patients with HFrEF when compared to those with HFmrEF and HFpEF. Previous studies have also shown that costs are higher in HFrEF than in HFpEF [48, 49]. Although some authors have suggested that this could be related with a higher risk of rehospitalizations, and a greater use of more invasive diagnostic procedures, more devices, such as implantable cardioverter defibrillator or resynchronization therapy, or even advanced support devices in patients with HFrEF compared to those with HFpEF [50, 51], others have observed that during the long-term follow-up these differences may reduce, particularly in those patients with HFpEF, presenting with more comorbidities [27]. In any case, the costs of HF hospitalization are substantial in patients with HFpEF [52]. Interestingly, the clinical profile, therapeutic approach, HCRU and costs were similar in the whole HFpEF spectrum, regardless of EF, suggesting that this is a homogeneous population. These data strongly suggest that to optimize the management of patients with HF, the approach may be quite different according to the type of HF. Thus, among patients with HFrEF, the introduction of disease-modifying therapies should started even during hospitalization after stabilization, and in those patients with HFpEF, not only the early introduction of SGLT2i should be encouraged, but also the appropriate treatment of comorbidities.

Polymedication was common in HF patients. This may lead to a lower medication adherence and a higher risk of drug-drug interactions [53]. As a result, those drugs that have demonstrated to modify the clinical course of HF should be considered. Unless contraindicated, guidelines recommend for patients with HFrEF the use of renin angiotensin system inhibitors (preferably sacubitril-valsartan), beta blockers, aldosterone antagonists and SGLT2i is mandatory [1]. In addition, different studies have shown that these drugs are also beneficial from a cost-effective point of view [54–56]. With regard to HFpEF, two recent clinical trials, the EMPEROR-Preserved and the DELIVER trials have shown that empagliflozin and dapagliflozin reduce the risk of the primary outcome among this population, respectively, particularly through a reduction of HF-related hospitalizations [15, 16] and this may lead to a marked reduction of HF burden, including HCRU and HF-related costs.

Our study also showed that HCRU and HF-related costs in HFmrEF patients were high, but in-between HFrEF and HFpEF. It has been reported that patients with HFmrEF have intermediate characteristics between HFrEF and HFpEF patients [22, 23, 25]. However, it is uncertain the best approach in these patients, and more information is warranted. In this setting, clinical trials, such as the DELIVER, that has included adults with symptomatic HF and EF > 40% and elevated natriuretic peptides, has provided important information about the best management in this population [16, 57].

Finally, indirect costs, mainly related with medical absenteeism, accounted for around 7% of total HF-related costs. Although due to the age of this patients, many of them would already be retired, as HF represents a substantial burden on the economy, productivity losses (indirect costs) should also be considered in the comprehensive management of patients with HF [58]. Remarkably, although this was not determined in our study, costs also should be analyzed from a social point of view, including the hours of dedication of the main caregiver or the professional who replaces him/her [58]. As a result, reducing the risk of HF (re-)hospitalizations, improving quality of life, as well as promoting an active working life should be considered as targets in the therapeutic approach of this population [1, 59].

Our study has some limitations. As this was an observational cohort study, using secondary data from electronic health records, only the information already collected in the electronic clinical history of patients could be recorded and, consequently some conditions may be underdiagnosed. In addition, to our knowledge, this is one of the first studies with a high number of patients assessing the HCRU and HF-related costs, with particular focus on EF subgroups in a nationally representative HF population.

In conclusion, HF is associated with high HCRU and direct and indirect healthcare costs across the whole EF spectrum. HF hospitalizations are responsible for nearly three quarters of HF-related costs, and HF medication represent less than 10% of total HF costs. Therefore, an optimization of HF therapy through a higher use of disease-modifying drugs could reduce disease and economic HF burdens. Although HF-related costs were higher among HFrEF, patients with HFmrEF and HFpEF patients represent a substantial burden, indicating that the optimization of treatment should be performed in the entire spectrum of HF, regardless of EF.

## Supplementary Information

**Additional file 1:** **Supplementary Table 1.** Definition of the variables (codes).

## Data Availability

The datasets used and/or analysed during the current study available from the corresponding author on reasonable request.
