# Supplementary material for: Healthcare resource utilization and costs among patients with heart failure with preserved, mildly reduced, and reduced ejection fraction in Spain
Source: BMC Health Serv Res. 2022 Oct 8;22:1241. doi: 10.1186/s12913-022-08614-x (PMC9547468; doi:10.1186/s12913-022-08614-x)
Supplement: Supplementary file 1 — Additional file 1: Supplementary Table 1. Definition of the variables (codes). [file 12913_2022_8614_MOESM1_ESM.docx]

**Supplementary table 1. Definition of the variables (codes).**

| **ICD-9** | **ICD-10** | **Description** |
| --- | --- | --- |
| 402.01, 402.11, 402.91 | I11.0 | Hypertensive heart disease with heart failure |
| 404.01, 404.11, 404.91 | I13.0 | Hypertensive heart and renal disease with (congestive) heart failure |
| 404.03, 404.13, 404.93 | I13.2 | Hypertensive heart and renal disease with both (congestive) heart failure and renal failure |
| 429.4, 997.1 | I97.1 | Other functional disturbances following cardiac surgery - Heart failure |
| 428.0 | I50.0 | Congestive Heart failure |
| 428.1 | I50.1 | Left ventricular failure, unspecified |
| 428.2 | I50.2 | Systolic heart failure |
| 428.3 | I50.3 | Diastolic heart failure |
| 428.4 | I50.4 | Combined systolic and diastolic heart failure |
| 428.9 | I50.9 | Heart failure, unspecified |
